# EHHADH deficiency regulates pexophagy and accelerates tubulointerstitial injury in diabetic kidney disease

**DOI:** 10.1038/s41420-024-02066-4

**Published:** 2024-06-15

**Authors:** Shuyan Kan, Qing Hou, Jinsong Shi, Mingchao Zhang, Feng Xu, Zhihong Liu, Song Jiang

**Affiliations:** National Clinical Research Center for Kidney Disease, Nanjing Jinling Hospital, Affiliated Hospital of Medical School, Nanjing University, Nanjing, China

**Keywords:** Pexophagy, Renal fibrosis

## Abstract

Peroxisomal l-bifunctional enzyme (EHHADH) plays a role in the classic peroxisomal fatty acid β-oxidation pathway; however, the relationship between EHHADH expression and diabetic kidney disease has not been well understood. Here, we found that endogenous EHHADH levels were strongly correlated with the progression and severity of diabetic nephropathy in T2D patients. EHHADH knockout mice exhibited worsened renal tubular injury in diabetic mice. Furthermore, EHHADH is a modulator of pexophagy. In renal tubular epithelial cells (RTECs) in vitro, the knockdown of EHHADH induced a dramatic loss of peroxisomes. The loss of peroxisomes in EHHADH-deficient RTECs was restored by either an autophagic inhibitor 3-methyladenine or bafilomycin A1 both in vitro and in vivo. NBR1 was required for pexophagy in EHHADH-knockdown cells, where the level of reactive oxygen species (ROS) was increased, while inhibition of ROS blocked pexophagy. In summary, our findings revealed EHHADH deficiency accelerated renal injury in DKD as a modulator of pexophagy.

## Introduction

Peroxisomes were first described in the 1960s [1]. Peroxisomes are ubiquitous organelles conserved across almost all eukaryotic cells and are single-membrane-bound intracellular organelles composed of cell membrane proteins and matrix proteins [2]. Because peroxisomes contain more than 50 different enzymes, they are very important metabolic platforms for purine catabolism, fatty acid oxidation (FAO), bile acid synthesis and ether phospholipid synthesis, including the catabolism of very long chain fatty acids, biosynthesis of ether-linked glycerolipids and bile acids synthesis [3]. Notably, peroxisomes play dual functions in the production and clearance of reactive oxygen species (ROS) and reactive nitrogen species [4, 5]. Peroxisomal dysfunction is linked to metabolic manifestations involved in diverse human diseases, such as neurodegenerative diseases [5], aging [6] and various kidney diseases [7, 8]. In humans, mutations in peroxin-encoding genes leading to the loss of peroxisomes cause peroxisome biogenesis disorders, such as Zellweger spectrum disorders [9], which can present as kidney impairment and dysfunction.

Peroxisome homeostasis is dynamically regulated by the interplay between peroxisome biogenesis and pexophagy, and pexophagy also contributes to the majority of peroxisome biogenesis disorders [10, 11]. Similar to mitophagy, pexophagy is selective macroautophagy/autophagy that targets peroxisomes [12]. The ubiquitination of peroxisomal membrane proteins (PMPs) or peroxisomal biogenesis factors (PEXs), such as PMP70 [13] and PEX5 [14], is required for pexophagy. Autophagy receptors, including SQSTM1 / p62, NBR1, NDP52, OPTN and TAX1BP1, are also capable of interacting with PMPs and sequestering target peroxisomes to autophagosomes for peroxisomal degradation [15]. In addition, PEX14 can directly interact with the LC3-II protein and mediate pexophagy [16].

Enoyl-CoA hydratase and 3-hydroxyacyl CoA dehydrogenase (EHHADH) is one of the enzymes involved in the peroxisomal FAO pathway [17]. EHHADH is mainly expressed in the liver and kidney. The mutation mistargets EHHADH delivery from the peroxisome to mitochondria, thereby disrupting mitochondrial metabolism and leading to renal Fanconi’s syndrome [18, 19]. EHHADH deficiency induces male-specific kidney hypertrophy without signs of severe kidney damage until 9 months after birth. However, in EHHADH-deficient mice, metabolic profiling analysis has suggested the occurrence of peroxisomal dysfunction [20]. A substantial loss of expression of peroxisomal proteins, including EHHADH, during acute kidney injury was identified in a recent proteomics analysis [21].

The role of EHHADH in the peroxisomal FAO pathway has been well-characterized [22, 23], but it remains poorly understood the implication of EHHADH for diabetic kidney disease. In the present study, we showed that the loss of EHHADH aggravated renal tubular injury in mice with diabetic kidney disease and resulted in a loss of peroxisomes in renal tubular epithelial cells (RTECs) as a novel pexophagy modulator.

## Results

### Renal tubular loss of EHHADH expression in T2D patients is correlated with diabetic nephropathy (DN) progression

**Fig. 1:**
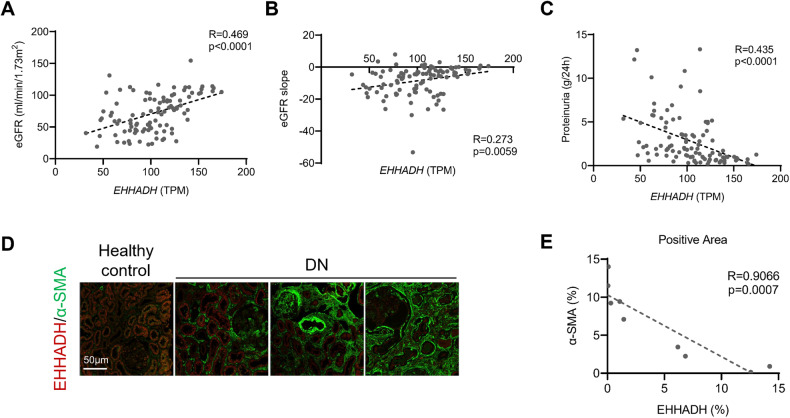
Renal tubular loss of *EHHADH* expression in patients is correlated with DN progression. **A** Association of tubular mRNA expression of *EHHADH* with eGFR determined by Spearman’s *R* test. **B** Association of tubular mRNA expression of *EHHADH* with eGFR slope determined by Spearman’s R test. **C** Association of tubular mRNA expression of *EHHADH* with 24 h proteinuria determined by Spearman’s *R* test. **D** Immunofluorescence staining of EHHADH (red) and α-SMA (green) in human kidney samples from healthy controls and patients with DN. **E** Association of EHHADH with α-SMA determined by Spearman’s *R* test.

To identify essential genes associated with renal tubulointerstitial injury in DN, we performed genome-wide gene expression profiling to figure out differentially expressed genes in renal tubulointerstitium between patients with DN and control donors (RPJNA666231 and GSE 158230). WGCNA of renal tubulointerstitium identified 18 gene coexpression modules (Supplementary Fig. 1A). Among these gene coexpression modules, the brown module, which includes 1136 transcribed genes (Table S1), exhibited the highest correlation with baseline eGFR (*R* = 0.7; *P* = 2 × 10^−20^). EHHADH was identified as one of the main hub genes in the Mebrown module combining MM and GS values (Supplementary Fig. 1B). Meanwhile, EHHADH was the only gene encoding a protein in in peroxisomes among the top 30 genes of the Mebrown module (Supplementary Table 1). Further analysis revealed that the expression level of EHHADH was positively correlated with eGFR (Fig. 1A) and eGFR slope (Fig. 1B), negatively correlated with the level of proteinuria (Fig. 1C) and tubulointerstitial fibrosis levels (Fig. 1D, E) in patients with DN. We further validated the expression patterns of *EHHADH* in the kidneys of our patients with DN (Fig. S1C) [24]. We also examined the expression of *EHHADH* in a public database to determine the relationship between EHHADH and kidney disease. The Nephroseq database (http://www.nephroseq.org) [25] showed that *EHHADH* mRNA expression was downregulated in the tubules of patients with DN (Fig. S1D); the estimated glomerular filtration rate (eGFR) level of patients with DN was significantly correlated with the expression levels of *EHHADH* in those patients (Fig. S1E). Single-cell transcriptional and chromatin accessibility profiling (http://humphreyslab.com/SingleCell/) [26] showed decreased expression and transcriptional activity of *EHHADH* in injured proximal tubules (Fig. S1F). Therefore, downregulation of *EHHADH* uniquely occurred in injured proximal tubules of patients with DN, and the endogenous expression of *EHHADH* was associated with the progression of DN.

### Genetic knockout of Ehhadh aggravates tubulointerstitial injury in diabetic mice

**Fig. 2:**
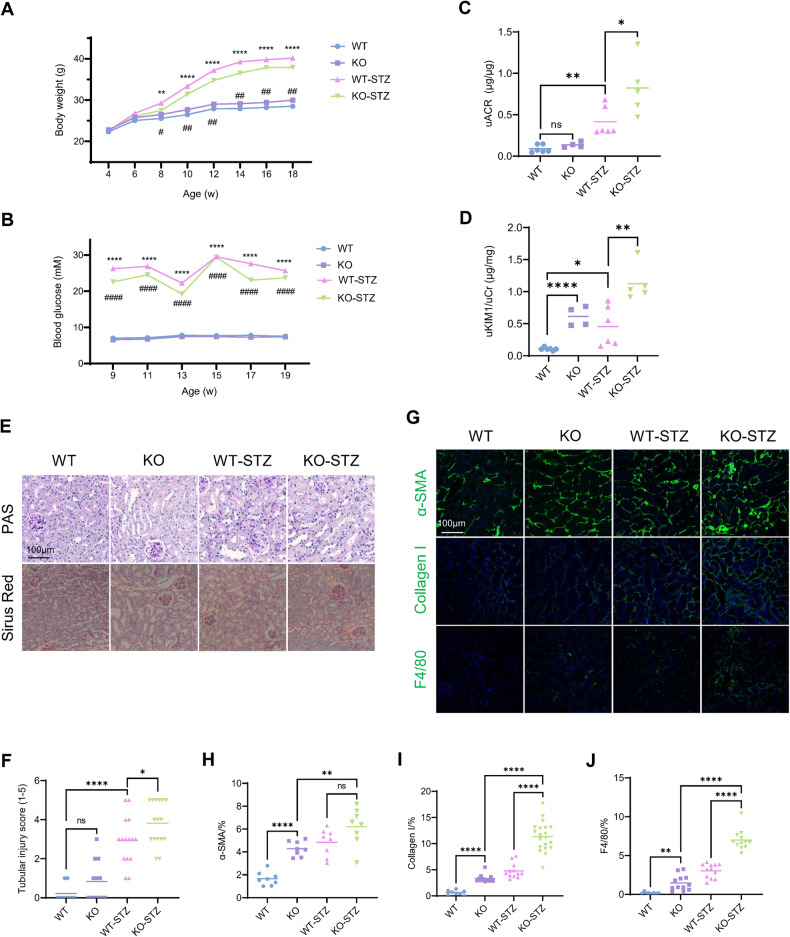
Genetic knockout of *Ehhadh* aggravates tubulointerstitial injury in diabetic mice. **A** Weight of WT, *Ehhadh* KO (KO), diabetic WT (WT-STZ) and diabetic *Ehhadh* KO (KO-STZ) mice. **B** Blood glucose levels of WT, KO, WT-STZ and KO-STZ mice. **C** Urine albumin-to-creatinine ratio (uACR) in WT, KO, WT-STZ, and KO-STZ mice. **D** Urine KIM1-to-creatinine ratio in WT, KO, WT-STZ, and KO-STZ mice. **E** Morphological examinations of kidney histology by periodic acid-Schiff (PAS) and Sirius red staining. **F** Quantification of tubular injury scoring in control and diabetic mice. **G** Representative images of IF staining of α-SMA, Collagen I and F4/80 in kidneys from control and diabetic mice. **H**–**J** Quantification of the positive areas of α-SMA, Collagen I and F4/80 in kidneys from control and diabetic mice. *p < 0.05, **p < 0.01, ***p < 0.001, ****p < 0.0001, ^#^*p* < 0.05, ^##^*p* < 0.01.

To examine whether EHHADH expression loss contributed to diabetic kidney disease development, diabetes was induced in male WT and *Ehhadh* KO mice (Fig. S2) by intraperitoneal injection of streptozotocin (STZ) after feeding them a high-fat diet (HFD). HFD-fed WT and *Ehhadh* KO mice exhibited increased body weights compared to normal chow-fed WT and *Ehhadh* KO mice after 4 weeks of the diet (Fig. 2A). The mice administered STZ developed higher blood glucose levels than control mice, indicating successful modeling of diabetes (Fig. 2B). There were no significant differences in body weight or blood glucose level between diabetic WT and diabetic *Ehhadh* KO mice. Compared to diabetic WT mice, diabetic *Ehhadh* KO mice developed more severe albuminuria (Fig. 2C), higher urine KIM1 (kidney injury molecule 1) level (Fig. 2D), worsened tubular injury, significant inflammatory filtrates and more severe interstitial fibrosis (Fig. 2E, F). In addition, IF staining of kidney tissues confirmed remarkable upregulation of α-SMA and Collagen I expression, as well as increased interstitial filtration of F4/80-positive macrophages (Fig. 2G–J) in diabetic *Ehhadh* KO mice compared with diabetic WT mice. Thus, a loss of *Ehhadh* expression aggravated tubulointerstitial injury under diabetic conditions.

### Knockdown of Ehhadh results in a loss of peroxisomes

**Fig. 3:**
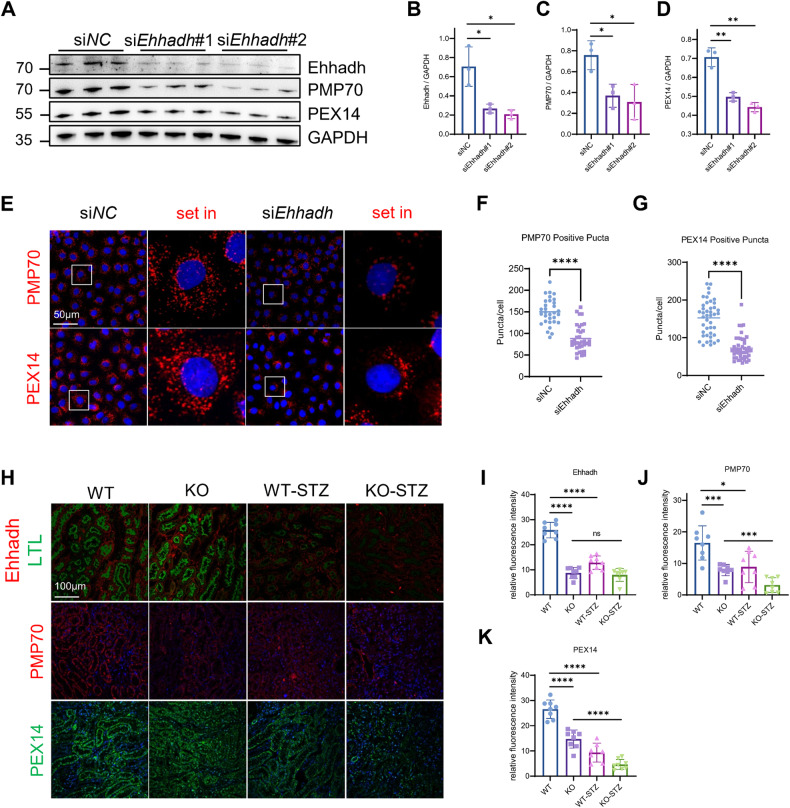
Knockdown of *Ehhadh* expression results in a loss of peroxisomes. **A** NRK-52E cells were transfected with scramble siRNA (si*NC*) or *Ehhadh*-targeting siRNA (si*Ehhadh*#1 and si*Ehhadh*#2), and the cells were harvested and analyzed by Western blotting with the indicated antibodies after 48 h of transfection. **B**–**D** Quantification of the relative protein expression levels of EHHADH, PMP70, and PEX14. **E** Immunofluorescence staining of PMP70 and PEX14 in NRK-52E cells transfected with si*NC* or si*Ehhadh*. **F** Quantification of PMP70-positive peroxisomes numbers per cell. **G** Quantification of PEX14-positive peroxisomes per cell. **H** Representative images of IF staining of EHHADH, PMP70, and PEX14 in kidneys from control and diabetic mice. **I**–**K** Quantification of the relative fluorescence intensity. *p < 0.05, **p < 0.01, ***p < 0.001, ****p < 0.0001.

First, high glucose treatment was performed in NRK-52E cells, a rat renal proximal tubular cell line. The expression level of Ehhadh decreased with the extension of high sugar treatment time (Fig. S3), which is consistent with our finding in T2D patients. We further investigated the consequences of EHHADH deficiency in NRK-52E cells. Two siRNA of *Ehhadh* (si*Ehhadh*#1 and si*Ehhadh*#2) was transfected in NRK-52E cells. The expression levels of both PEX14 and PMP70, markers of peroxisomes, were significantly decreased in *Ehhadh*-knockdown cells with different si*Ehhadh* transfected, compared with scramble cells (Fig. 3A–D). Confocal microscopy results also showed the dramatic loss of PEX14 and PMP70 puncta in *Ehhadh*-knockdown cells. Notably, peroxisomes exhibited perinuclear distribution inhibition after the loss of EHHADH (Fig. 3E–G). In vivo, *Ehhadh* KO mice displayed a clear reduction in PMP70 and PEX14 expression in RETCs compared with WT mice, which was more pronounced in diabetic *Ehhadh* KO and WT mice (Fig. 3H–K). In summary, the results suggest that inhibition of EHHADH induces a loss of peroxisomes in RETCs.

### EHHADH deficiency enhances pexophagy

**Fig. 4:**
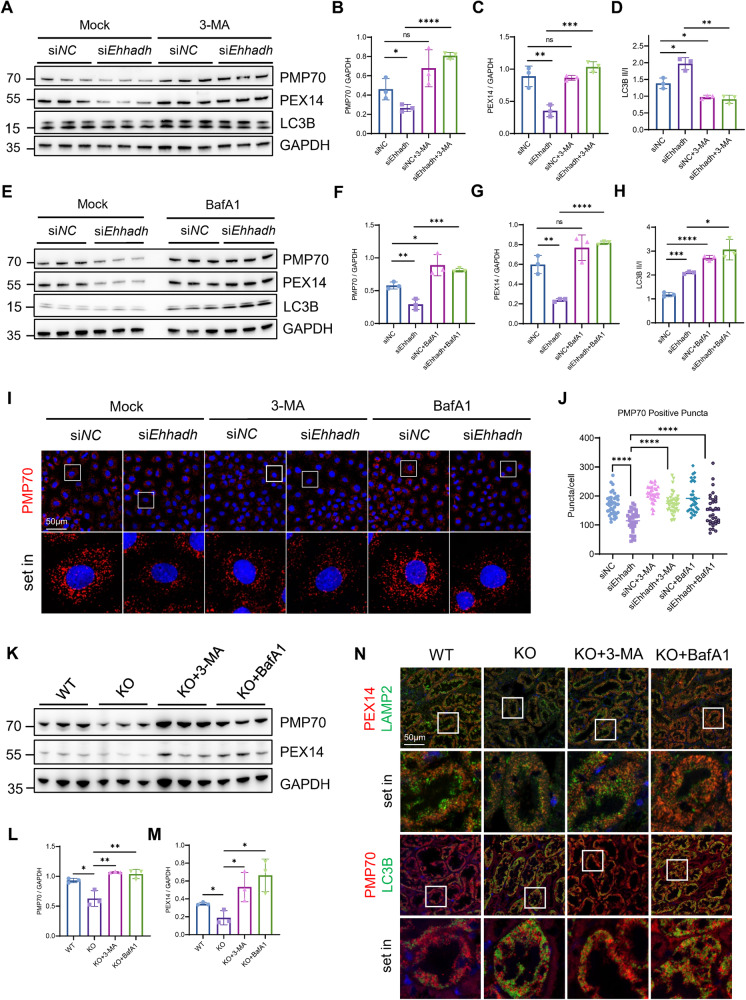
Deficiency of *Ehhadh* expression enhances pexophagy in renal tubular cells both in vitro and in vivo. **A** NRK-52E cells were transfected with si*NC* or si*Ehhadh*. The cells were treated with 3-MA (5 mM) for 6 h and then harvested at 48 h after transfection and analyzed by Western blotting with the indicated antibodies. **B**–**D** Quantification of the relative protein expression levels of PMP70 and PEX14 and the LC3B II/I ratio. **E** NRK-52E cells were transfected with si*NC* or si*Ehhadh*. Prior to 48 h, the cells were treated with BafA1 (500 nM) for 6 h and then harvested and analyzed by Western blotting with the indicated antibodies. **F**–**H** Quantification of the relative protein expression levels of PMP70 and PEX14 and the LC3B II/I ratio. **I** Immunofluorescence staining of PMP70 in NRK-52E cells transfected with si*NC* or si*Ehhadh* for 48 h and treated with or without 3-MA or BafA1. **J** Quantification of PMP70-positive peroxisome numbers per cell. **K** Immunoblotting analysis of kidney protein expression levels in mice treated with or without 3-MA or BafA1. **L**, **M** Quantification of the relative protein expression levels of PMP70 and PEX14. **N** Representative IF staining images of PEX14 and LAMP2, PMP70 and LC3B in the renal tubules of WT or KO mice treated with or without 3-MA or BafA1. *p < 0.05, **p < 0.01, ***p < 0.001, ****p < 0.0001.

We speculate that both peroxisome biogenesis disorder and enhanced pexophagy may account for the loss of peroxisomes. Peroxisome proliferator-activated receptors (PPARs) are critical for modulating the transcriptional activity of genes involved in peroxisome biogenesis [27, 28]. In agreement, Bezafibrate, a PPAR agonist, reversed the downregulation of PEX14 and PMP70 in *Ehhadh*-knockdown cells, (Fig. S4A–E). As mentioned above, the perinuclear distribution of peroxisome markers led us to hypothesize that there might be colocalization of peroxisomes and lysosomes due to enhanced pexophagy. Thus, we further investigated the role of pexophagy in *Ehhadh*-knockdown cells. The loss of PMP70 and PEX14 expression in *Ehhadh*-knockdown cells was significantly restored by both an autophagic inhibitor (3-methyladenine, 3-MA) (Fig. 4A–D) and a lysosome fusion inhibitor (bafilomycin A1, BafA1) (Fig. 4E–H), along with the restoration of peroxisomal puncta (Figs. 2I, J, S4F, G), suggesting that inhibition of EHHADH increased pexophagy, leading to the degradation of peroxisomes. Confocal imaging analysis confirmed the clear colocalization of peroxisomes with autophagosomes and lysosomes in *Ehhadh*-knockdown cells, but this colocalization was prohibited by 3-MA or BafA1 (Fig. S4H).

To confirm our findings in vitro, we compared the numbers of peroxisomes present in the kidneys of WT mice to those of *Ehhadh* KO mice with or without 3-MA or BafA1 treatment. Correspondingly, Western blotting results showed decreased protein expression levels of both PMP70 and PEX14 in the kidneys of *Ehhadh* KO mice compared to those in the kidneys of WT mice. After 3 consecutive days of treatment with 3-MA or BafA1, the protein expression levels of both PMP70 and PEX14 were remarkably rescued (Fig. 4K–M). Importantly, we observed the colocalization of peroxisome markers with autophagosomes and lysosomes in mouse renal tubular cells (Fig. 4N). The loss of peroxisomes in RETCs was associated with enhanced pexophagy in diabetic model mice and enhanced in diabetic Ehhadh-KO mice as well (Fig. S4I). In summary, EHHADH regulates pexophagy in RTECs.

### Levels of ROS increase in Ehhadh-knockdown cells

**Fig. 5:**
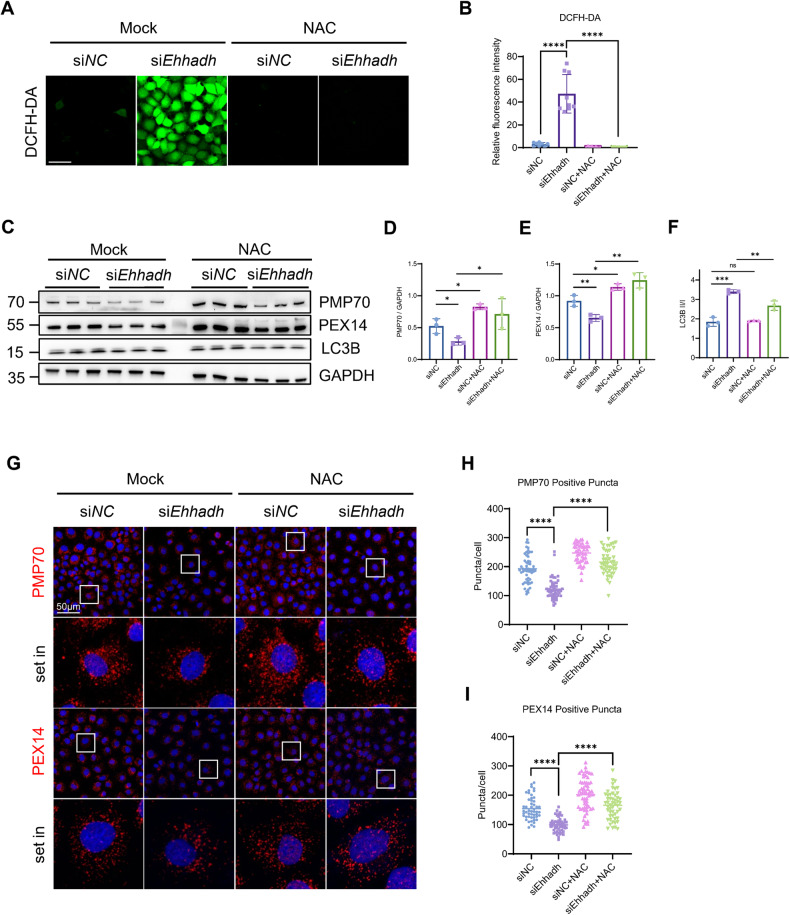
EHHADH-mediated pexophagy primarily depends on ROS levels. **A** NRK-52E cells were transfected with si*NC* or si*Ehhadh*. After 24 h, the cells were treated with NAC (1 mM) for 24 h and then incubated with DCFH-DA. **B** Quantification of the relative fluorescence intensity. **C** NRK-52E cells were transfected with si*NC* or si*Ehhadh*. After 24 h, the cells were treated with NAC (1 mM) for 24 h and then harvested and analyzed by Western blotting with the indicated antibodies. **D**–**F** Quantification of the relative protein expression levels of PMP70 and PEX14 and the LC3B II/I ratio. **G** Immunofluorescence staining of PMP70 and PEX14 in NRK-52E cells transfected with si*NC* or si*Ehhadh* for 48 h and treated with or without NAC. **H** Quantification of PMP70-positive peroxisomes per cell. **I** Quantification of PEX14-positive peroxisomes per cell. *p < 0.05, **p < 0.01, ***p < 0.001, ****p < 0.0001 .

Increased reactive oxygen species levels can induce pexophagy [29], and we sought to determine whether pexophagy in EHHADH-knockdown cells was induced by ROS production. In NRK-52E cells, inhibition of EHHADH greatly increased ROS levels, which were completely blocked by administration of N-acetylcysteine (NAC), a ROS inhibitor (Fig. 5A, B). Immunoblotting (Fig. 5C–F) and confocal imaging (Fig. 5G–I) analysis demonstrated that NAC treatment restored the numbers of peroxisomes in *Ehhadh*-knockdown cells. Consequently, we concluded that the loss of EHHADH induced pexophagy by enhancing ROS levels.

### NBR1 is required for pexophagy in Ehhadh-knockdown cells

**Fig. 6:**
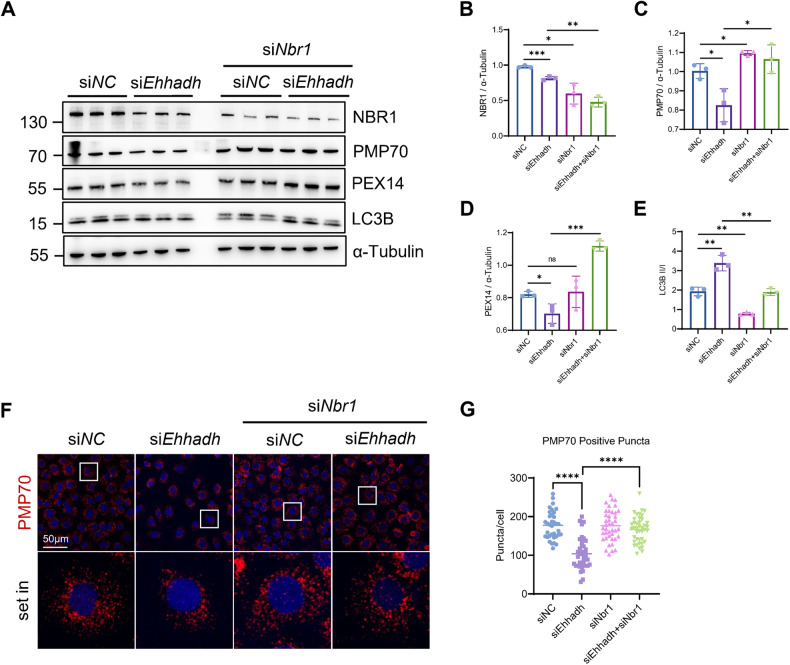
NBR1 is required for pexophagy in *Ehhadh*-knockdown cells. **A** NRK-52E cells were transfected with si*NC* or si*Ehhadh* in the presence or absence of *Nbr1*-targeting siRNA (si*Nbr1*). After 48 h, the cells were harvested and analyzed by Western blotting with the indicated antibodies. **B–E** Quantification of the relative protein expression levels of PMP70, PEX14 and NBR1 and the LC3B II/I ratio. **F** Immunofluorescence staining of PMP70 and PEX14 in NRK-52E cells transfected with si*NC* or si*Ehhadh* in the presence or absence of si*Nbr1*. **G** Quantification of PMP70-positive peroxisomes per cell. *p < 0.05, **p < 0.01, ***p < 0.001, ****p < 0.0001.

NBR1 functions as an important autophagic receptor in the process of pexophagy [30], and we investigated whether NBR1 was involved in EHHADH-mediated pexophagy. NBR1 was downregulated in *Ehhadh*-knockdown cells (Fig. S5A, B). Further, inhibition of NBR1 by siRNA in *Ehhadh*-knockdown cells markedly restored the loss of PMP70 and PEX14 expression levels (Fig. 6A–E). The number of peroxisomal puncta was rescued after si*Nbr1* transfection (Fig. 6F, G). The ubiquitination of PMP70 was required for pexophagy [13]. In Myc-PMP70- and HA-Ubiquitin-K63-transfected HEK293T cells, knockdown of *EHHADH* levels resulted in an increased ubiquitination level of Myc-PMP70 and enhanced binding to NBR1 (Fig. S5E). Collectively, our results suggest that NBR1 is required for pexophagy in EHHADH-knockdown cells.

## Discussion

In the current study, we identified EHHADH as a critical gene correlated with eGFR by integrating renal biopsy specimen-derived transcriptional profiles from DN patients. We also showed that genetic loss of *Ehhadh* expression aggravated tubulointerstitial injury under diabetic conditions. In mechanism, the inhibition of EHHADH expression led to a loss of peroxisomes in RTECs through pexophagy, which is relative to the increased ROS levels. Additionally, the autophagic receptor NBR1 was required for EHHADH-mediated pexophagy.

Tubular injury is a key factor in the development and progression of DN [31]. Abnormal lipid metabolism and oxidative stress are one of the main causes of tubular injury mediated in high glucose condition [32–34]. Peroxisomes are intimately associated with lipid metabolism and oxidative stress, and their ability to carry out fatty acid oxidation and lipid synthesis, especially the production of ether lipids, may be critical for generating cellular signals required for normal physiology. Peroxisomes are highly enriched in kidneys, and a reduced abundance of functional peroxisomes has been observed in RTECs from both human patient kidney samples and in several experimental models of kidney diseases [7, 8, 35, 36]. Previous studies have also found that peroxisome is downregulated in a variety of kidney diseases. PEX5 and PEX11a are both the essential functional proteins of peroxisomes. Renal tubular conditional deletion of *Pex5* (*Pex5*^cKO^) [37], or knockout *Pex11a* [38] in mice resulted in dramatic loss of peroxisomes, but not affect the kidney survival. However, integrated transcriptomic and metabolomic analyses showed profound reprogramming in the metabolic, antioxidant and lipid synthesis pathways of the kidneys of *Pex5*^cKO^ mice. The result was in line with our findings in *Ehhadh* KO mice. We speculate that loss of *EHHADH* expression increases susceptibility to the development of DN, and analysis from the public database showed that EHHADH expression was specifically downregulated in damaged tubules of patients with DN [26]. As expected, although the weight and blood glucose of *Ehhadh* KO mice were slightly lower than those of WT mice under diabetic conditions, they still displayed more severe tubulointerstitial injury, indicating the importance of EHHADH in tubulointerstitial injury under hyperglycemia, which also is in agreement with previous findings that PEX11a deficiency aggravated renal interstitial lesions in mice [39].

A previous study established the association between EHHADH expression and renal tubular injury in the kidneys of male mice, which had peroxisomal dysfunction and related metabolite changes, but the underlying mechanism is not well explained [20]. The p.E3K mutation in EHHADH was identified in five generations of a family with isolated autosomal dominant Fanconi renal syndrome. The p.E3K mutation caused mistargeting of peroxisomal EHHADH to mitochondria, leading to impaired mitochondrial fatty acid β-oxidation and respiration and decreased ATP production, but peroxisomal deficiency and dysfunction were not observed [18, 19].

Based on our above findings, we confirmed that a lack of peroxisomal EHHADH expression causes peroxisomal deficiency and dysfunction. Similar to mitophagy, peroxisomes can be targeted for selective autophagic degradation in lysosomes, which is called pexophagy [40, 41]. This is the first study to present the relationship between EHHADH expression and pexophagy. EHHADH plays an important role in peroxisomal FAO metabolism; indeed, we observed that *Ehhadh* knockdown in RTECs increased ROS levels, which served as the inducer of pexophagy. Peroxisomes damage leads to increased intracellular ROS, which can also induce increased pexophagy. Our study also observed increased intra cell ROS in *Ehhadh* knockdown RTECs. In addition, the increased pexophagy caused by EHHADH downregulation was significantly restored after ROS inhibition, indicating that EHHADH-mediated pexophagy is partially dependent on ROS production.

The ubiquitination of PMPs is required for pexophagy [42–44], and NBR1 was shown to play an important role in EHHADH-mediated pexophagy. Although we observed increased ubiquitination of PMP70 and enhanced binding between PMP70 and NBR1 in EHHADH-knockdown RTECs, the precise binding sites and other PMPs that underwent ubiquitination and coordinated with NBR1 still need further study.

In summary, our study has provided novel insights into the regulation of pexophagy by EHHADH deficiency. We have identified that downregulation of EHHADH can lead to peroxisomal deficiency, which in turn increases the susceptibility to tubular injury and fibrosis under hyperglycemia. These findings highlight the potential of targeting pexophagy as a therapeutic strategy for the treatment and prevention of DN in the future.

## Materials and methods

### Antibodies and reagents

The antibodies used were as follows: EHHADH (bs-4058R, Bioss Antibodies), ABCD3 (Affinity Biosciences, DF831), PEX14 (Proteintech Group, 105494-1-AP), LC3 (Cell Signaling Technology, 12741S), NBR1 (Proteintech Group, 16004-1-AP), α-tubulin (Proteintech Group, 66031-1-Ig), and GAPDH (Proteintech Group, 60004-1-Ig). HRP-conjugated goat anti-rabbit IgG (Boster Biological Technology, BA1054) and HRP-conjugated goat anti-mouse IgG (Boster Biological Technology, BA105) were used for Western blotting analysis. PMP70 (Abcam, AB3421), PEX14 (Proteintech Group, 105494-1-AP), Lamp2 (Servicebio Technology, GB11330), EHHADH (Santa Cruz Biotechnology, sc-393123), α-SMA (Proteintech Group, 14395-1-AP), COL1A1 (Cell Signaling Technology, 72026), F4/80 (Cell Signaling Technology, 70076 S), LC3B (Proteintech Group, 18725-1-AP), LTL (Vector Laboratories, FL-1321), Alexa Fluor 555 donkey anti-rabbit (Thermo Fisher Scientific, A31572), Alexa Fluor 488 donkey anti-mouse (Thermo Fisher Scientific, A21202), and Alexa Fluor 488 donkey anti-rabbit (Thermo Fisher Scientific, A21206) antibodies were used for immunofluorescence staining.

### Human kidney samples

The kidney tissues of patients with biopsy-proven DN and healthy controls used in this study were obtained from the Renal Biobank of National Clinical Research Center of Kidney Diseases, Jiangsu Biobank of Clinical Resources. The clinical characteristics of patients with DN have been described previously [24].

### Renal tubulointerstitial weighted gene coexpression network analysis (WGCNA)

Renal tubulointerstitial transcripts profile is deposited at Sequence Read Archive (www.ncbi.nlm.nih.gov/sra/) and Gene Expression Omnibus (www.ncbi.nlm.nih.gov/geo/) under reference nos. PRJNA666231 and GSE158230, respectively. Weighted gene coexpression network analysis (WGCNA) was used to cluster coexpressed genes (Gene module). The WGCNA was constructed by using the WGCNA package in R as described before [45].

### Mice and diabetic modeling

C57BL/6N-*Ehhadh*^em1C^/Cya (KOCMP-74147-*Ehhadh*-B6N-VA) mice were generated by Cyagen Biosciences Inc. (Suzhou, China). All mice (4-6 mice per cage) were housed under standard laboratory conditions. The primers used for genotyping are listed in Table S2. For autophagy inhibitor treatment, *Ehhadh* KO mice *(n* = 3) received an intraperitoneal injection of 10 mg/kg/d 3-MA or 1 mg/kg/d BafA1. The mice were sacrificed after 3 days of 3-MA or BafA1 treatment; WT and *Ehhadh* KO mice served as normal controls. A diabetic model was induced with HFD combined with continuous low-dose STZ treatment. WT and *Ehhadh* KO male mice (*n* = 6) were fed a high-fat diet (Dyets, HF60) beginning at 4 weeks of age, followed by intraperitoneal injection of 50 mg/kg/d STZ for 5 days to induce diabetes at 8 weeks of age. A vehicle was injected into WT and *Ehhadh* KO mice (*n* = 6) fed with control diet, which served as a normal control. WT or KO mice were randomly allocated to control or diabetic group. One week after the STZ injection, mice with blood glucose greater than 16.7 mmol/L were included in subsequent experiments. The mice were sacrificed 12 weeks after STZ injection. The Institutional Animal Care and Use Committee of Jinling Hospital, Nanjing University School of Medicine approved the experiments.

### Urinary albumin, KIM1, and creatinine assay

The levels of murine urinary albumin, KIM1 and creatinine were measured using an Albumin ELISA Kit (Genetex, GTX37052), a Mouse KIM1 ELISA Kit (NEOBIOSCIENCE, EMC018) and a Creatinine Assay Kit (BioAssay Systems, DICT-500) following the manufacturer’s instructions.

### Mouse kidney histology and IF staining

Murine kidneys were removed and fixed in 4% PFA at 4 °C overnight followed by periodic acid-Schiff (PAS) and Sirius Red staining. The tubular injury score was measured according to the standard described previously [38]. For IF staining, sectioned paraffin-embedded kidney tissue was deparaffinized and hydrated. Heat-induced epitope retrieval was employed. Tissue sections were incubated with the indicated primary antibodies overnight at 4 °C after blocking in 10% FBS for 20 min at room temperature. After 2 h of incubation with secondary antibodies at room temperature in the dark, tissue sections were mounted with DAPI for confocal microscope imaging.

### Plasmids and siRNAs

Expression plasmid of pcDNA3.1 + N-Myc-PMP70 was synthesized by GenScript Company (Nanjing, China). pRK5-HA-Ubiquitin-K63 (P19710) was purchased from MiaoLing Plasmid Platform. Short interfering RNAs (siRNAs) targeting human *EHHADH*, rat *Ehhadh* and rat *Nbr1* were synthesized by RiboBio Company (Guangzhou, China). Both si*Ehhadh*#1 and si*Ehhadh*#2 are effective and siEhhadh#2 was applied in the follow-up experiments. The sequences of the siRNAs used are listed in Table S2.

### Cell culture and transfection

NRK-52E and human embryonic kidney (HEK293T) cell lines were purchased from the American Type Culture Collection (ATCC). NRK-52E cells were maintained in Dulbecco’s modified Eagle’s medium-F12 medium (DMEM-F12, Gibco C11330500) plus 5% fetal bovine serum (FBS, CELLiGent CG0430B) and 1% (v/v) penicillin‒streptomycin (NCM Biotech) at 37 °C in a 5% carbon dioxide atmosphere. HEK293T cells were grown in Dulbecco’s modified Eagle’s medium (DMEM) (VivaCell, C3114-0500) supplemented with 10% FBS + 1% penicillin/streptomycin. Plasmids and siRNAs were transfected into NRK-52E or HEK293T cells using jetPRIME DNA / siRNA transfection reagent (Polyplus, Germany) according to the manufacturer’s instructions. Cells were harvested for analysis after 48 h of transfection.

### Immunoblotting analysis

Protein from NRK-52E cells and mouse kidneys was lysed with M-PER^TM^ Mammalian Protein Extraction Reagent (Thermo Scientific) supplemented with a protease inhibitor cocktail (Roche). The protein concentration was determined by BCA (Beyotime, P0012). Equal amounts of protein samples were loaded and separated by 4–20% Bis-Tris Gel (Future, F11420Gel) and then transferred onto polyvinylidene-fluoride membranes (PVDF) (Merck, IPVH00010). PVDF membranes were blocked with 5% nonfat milk in PBST for 1 h at room temperature and then incubated with the indicated primary antibodies overnight at 4 °C with rotation. After several washes with PBST, HRP-conjugated secondary antibodies were added for 1 h at room temperature. An NCM ECL Ultra kit (NCM Biotech, P10200) was used for signal detection.

### Coimmunoprecipitation (Co-IP)

Cell lysates within M-PER^TM^ Mammalian Protein Extraction Reagent supplemented with the protease inhibitor cocktail (Roche) at 24 h after transfection in HEK293T cells were immunoprecipitated with anti-Myc magnetic beads overnight at 4 °C with gentle rotation. After five washes with cold IP buffer, the immune complexes were eluted in 2 × SDS buffer and subjected to immunoblotting analysis.

### IF staining in cells

NRK-52E cells were plated in glass bottom cell culture dishes (NEST Scientific, 801002). After three washes with PBS, cells were fixed in 4% PFA for 20 min and permeabilized with PBS containing 0.5% Triton-X100 for 10 min at room temperature. After blocking with 10% FBS in PBS for 30 min at room temperature, the cells were incubated with the indicated primary antibodies overnight at 4 °C, followed by secondary antibody incubation for 2 h at room temperature in the dark. Cells were mounted with DAPI for confocal microscope imaging (Zeiss LSM900, Germany). The numbers of puncta were quantified by ImageJ FIJI software.

### ROS measurement

Redox levels were measured with dichlorodihydrofluorescein diacetate (DCFH-DA) (S0033S, Beyotime, China). NRK-52E cells were treated with or without NAC for 24 h after transfection with siRNA. Cells were treated with 10 μmol/L DCFD-DA at 37 °C in the dark for 30 min and then washed with PBS 3 times. Live cell fluorescence was imaged by confocal microscopy. The mean fluorescence intensity was measured with ImageJ FIJI.

### Statistical analysis

All data are presented as the mean ± SD of three independent experiments. Analysis of variance was performed and assumption criteria were met and analysis of variance was performed. Statistical comparisons of two groups for a single variable with normal distributions were analyzed by unpaired *t*-test. Statistical comparisons of multi-group with one independent variable were analyzed by One-way ANOVA, with a *p* value < 0.05 considered statistically significant. All statistical analyses employed were performed using GraphPad Prism 9.

### Supplementary information

Supplemental Figures

Supplementary Table 1

Supplementary Table 2

Original data of WB

## Data Availability

The data used and/or analyzed during the study are available from the corresponding author on reasonable request.

## References

[1] De Duve C, Baudhuin P (1966). Peroxisomes (microbodies and related particles). Physiol Rev.

[2] Wanders RJA, Baes M, Ribeiro D, Ferdinandusse S, Waterham HR (2023). The physiological functions of human peroxisomes. Physiol Rev.

[3] He A, Dean JM, Lodhi IJ (2021). Peroxisomes as cellular adaptors to metabolic and environmental stress. Trends Cell Biol.

[4] Fransen M, Nordgren M, Wang B, Apanasets O (2012). Role of peroxisomes in ROS/RNS-metabolism: implications for human disease. Biochim Biophys Acta.

[5] Griffey CJ, Yamamoto A (2022). Macroautophagy in CNS health and disease. Nat Rev Neurosci.

[6] Terlecky SR, Koepke JI, Walton PA (2006). Peroxisomes and aging. Biochim Biophys Acta.

[7] Vasko R (2016). Peroxisomes and kidney injury. Antioxid Redox Signal.

[8] Shamekhi Amiri F (2019). Intracellular organelles in health and kidney disease. Nephrol Ther.

[9] Braverman NE, Raymond GV, Rizzo WB, Moser AB, Wilkinson ME, Stone EM (2016). Peroxisome biogenesis disorders in the Zellweger spectrum: An overview of current diagnosis, clinical manifestations, and treatment guidelines. Mol Genet Metab.

[10] Fujiki Y, Okumoto K, Honsho M, Abe Y (2022). Molecular insights into peroxisome homeostasis and peroxisome biogenesis disorders. Biochim Biophys Acta Mol Cell Res.

[11] Nazarko TY (2017). Pexophagy is responsible for 65% of cases of peroxisome biogenesis disorders. Autophagy.

[12] Germain K, Kim PK (2020). Pexophagy: a model for selective autophagy. Int J Mol Sci.

[13] Zheng J, Chen X, Liu Q, Zhong G, Zhuang M (2022). Ubiquitin ligase MARCH5 localizes to peroxisomes to regulate pexophagy. J Cell Biol.

[14] Demers ND, Riccio V, Jo DS, Bhandari S, Law KB, Liao W (2023). PEX13 prevents pexophagy by regulating ubiquitinated PEX5 and peroxisomal ROS. Autophagy.

[15] Cho DH, Kim YS, Jo DS, Choe SK, Jo EK (2018). Pexophagy: molecular mechanisms and implications for health and diseases. Mol Cells.

[16] Zutphen T, Veenhuis M, van der Klei IJ (2008). Pex14 is the sole component of the peroxisomal translocon that is required for pexophagy. Autophagy.

[17] Zhao S, Xu W, Jiang W, Yu W, Lin Y, Zhang T (2010). Regulation of cellular metabolism by protein lysine acetylation. Science.

[18] Klootwijk ED, Reichold M, Helip-Wooley A, Tolaymat A, Broeker C, Robinette SL (2014). Mistargeting of peroxisomal EHHADH and inherited renal Fanconi’s syndrome. N. Engl J Med.

[19] Assmann N, Dettmer K, Simbuerger JMB, Broeker C, Nuernberger N, Renner K (2016). Renal Fanconi Syndrome Is Caused by a Mistargeting-Based Mitochondriopathy. Cell Rep.

[20] Ranea-Robles P, Portman K, Bender A, Lee K, He JC, Mulholland DJ (2021). Peroxisomal L-bifunctional protein (EHHADH) deficiency causes male-specific kidney hypertrophy and proximal tubular injury in mice. Kidney360.

[21] Burton JB, Silva-Barbosa A, Bons J, Rose J, Pfister K, Simona F, et al. Substantial downregulation of mitochondrial and peroxisomal proteins during acute kidney injury revealed by data-independent acquisition proteomics. bioRxiv. 2023. 10.1101/2023.02.26.530107. 10.1002/pmic.202300162 37775337

[22] Houten SM, Denis S, Argmann CA, Jia Y, Ferdinandusse S, Reddy JK (2012). Peroxisomal L-bifunctional enzyme (Ehhadh) is essential for the production of medium-chain dicarboxylic acids. J Lipid Res.

[23] Yang G, Sun S, He J, Wang Y, Ren T, He H (2023). Enoyl-CoA hydratase/3-hydroxyacyl CoA dehydrogenase is essential for the production of DHA in zebrafish. J Lipid Res.

[24] Hou Q, Kan S, Wang Z, Shi J, Zeng C, Yang D (2022). Inhibition of HDAC6 with CAY10603 ameliorates diabetic kidney disease by suppressing NLRP3 inflammasome. Front Pharmacol.

[25] Ju, Nair W, Smith V, Zhu S, Shedden L, Song PXK K (2015). Tissue transcriptome-driven identification of epidermal growth factor as a chronic kidney disease biomarker. Sci Transl Med.

[26] Wilson PC, Muto Y, Wu H, Karihaloo A, Waikar SS, Humphreys BD (2022). Multimodal single cell sequencing implicates chromatin accessibility and genetic background in diabetic kidney disease progression. Nat Commun.

[27] Crouser ED (2010). Peroxisome proliferator-activated receptors gamma coactivator-1alpha: master regulator of mitochondrial biogenesis and survival during critical illness?. Am J Respir Crit Care Med.

[28] Pawlak M, Lefebvre P, Staels B (2015). Molecular mechanism of PPARα action and its impact on lipid metabolism, inflammation and fibrosis in non-alcoholic fatty liver disease. J Hepatol.

[29] Jo DS, Park SJ, Kim AK, Park NY, Kim JB, Bae JE (2020). Loss of HSPA9 induces peroxisomal degradation by increasing pexophagy. Autophagy.

[30] Deosaran E, Larsen KB, Hua R, Sargent G, Wang Y, Kim S (2013). NBR1 acts as an autophagy receptor for peroxisomes. J Cell Sci.

[31] Zhou X, Xu C, Dong J, Liao L (2023). Role of renal tubular programed cell death in diabetic kidney disease. Diab Metab Res Rev.

[32] Mori Y, Ajay AK, Chang JH, Mou S, Zhao H, Kishi S (2021). KIM-1 mediates fatty acid uptake by renal tubular cells to promote progressive diabetic kidney disease. Cell Metab.

[33] Egbujor MC, Petrosino M, Zuhra K, Saso L (2022). The role of organosulfur compounds as Nrf2 activators and their antioxidant effects. Antioxidants.

[34] Sun Y, Cui S, Hou Y, Yi F (2021). The updates of podocyte lipid metabolism in proteinuric kidney disease. Kidney Dis.

[35] Yoshioka K, Hirakawa Y, Kurano M, Ube Y, Ono Y, Kojima K (2022). Lysophosphatidylcholine mediates fast decline in kidney function in diabetic kidney disease. Kidney Int.

[36] Dhillon P, Park J, Hurtado Del Pozo C, Li L, Doke T, Huang S (2021). The nuclear receptor ESRRA protects from kidney disease by coupling metabolism and differentiation. Cell Metab.

[37] Ansermet C, Centeno G, Pradervand S, Harmacek D, Garcia A, Daraspe J (2022). Renal tubular peroxisomes are dispensable for normal kidney function. JCI Insight.

[38] Chen J, Chen JK, Conway EM, Harris RC (2013). Survivin mediates renal proximal tubule recovery from AKI. J Am Soc Nephrol.

[39] Weng H, Ji X, Endo K, Iwai N (2014). Pex11a deficiency is associated with a reduced abundance of functional peroxisomes and aggravated renal interstitial lesions. Hypertension.

[40] Dunn WA, Cregg JM, Kiel JA, van der Klei IJ, Oku M, Sakai Y (2005). Pexophagy: the selective autophagy of peroxisomes. Autophagy.

[41] Vargas JNS, Hamasaki M, Kawabata T, Youle RJ, Yoshimori T (2023). The mechanisms and roles of selective autophagy in mammals. Nat Rev Mol Cell Biol.

[42] Li J, Wang W (2021). Mechanisms and functions of pexophagy in mammalian cells. Cells.

[43] Zhang J, Tripathi DN, Jing J, Alexander A, Kim J, Powell RT (2015). ATM functions at the peroxisome to induce pexophagy in response to ROS. Nat Cell Biol.

[44] Yamashita S, Abe K, Tatemichi Y, Fujiki Y (2014). The membrane peroxin PEX3 induces peroxisome-ubiquitination-linked pexophagy. Autophagy.

[45] Pan Y, Jiang S, Hou Q, Qiu D, Shi J, Wang L (2018). Dissection of glomerular transcriptional profile in patients with diabetic nephropathy: SRGAP2a protects podocyte structure and function. Diabetes.

