# Supplementary material for: EHHADH deficiency regulates pexophagy and accelerates tubulointerstitial injury in diabetic kidney disease
Source: Cell Death Discov. 2024 Jun 15;10:289. doi: 10.1038/s41420-024-02066-4 (PMC11180138; doi:10.1038/s41420-024-02066-4)

**Figure S1. EHHADH expression in human diabetic kidneys.** (A) Weighted gene co-expression network analysis (WGCNA) is performed and each module is assigned a unique color label. Brown module is significantly associated with eGFR (R = 0.7; P =2×10^-20^). (B) A scatterplot of Gene Significance (GS) for weight vs. Module Membership (MM) in the brown module. Point of EHHADH is highlighted in red. (**C**) The mRNA expression of tubular EHHADH in patients with different stages of DN in our cohort. (**D**) The mRNA expression of tubular EHHADH from patients with various kidney diseases from the Ju study cohort. (**E**) Association of tubular mRNA expression of EHHADH with eGFR in patients with DN determined by Spearman’s R test. (**F**) Decreased expression and transcriptional activity of EHHADH in injured proximal tubules from patients with DN. Two-tailed Student’s t test, *p < 0.05, ***p < 0.001, ****p < 0.0001, ns: not significant. Values are the means ± SDs.


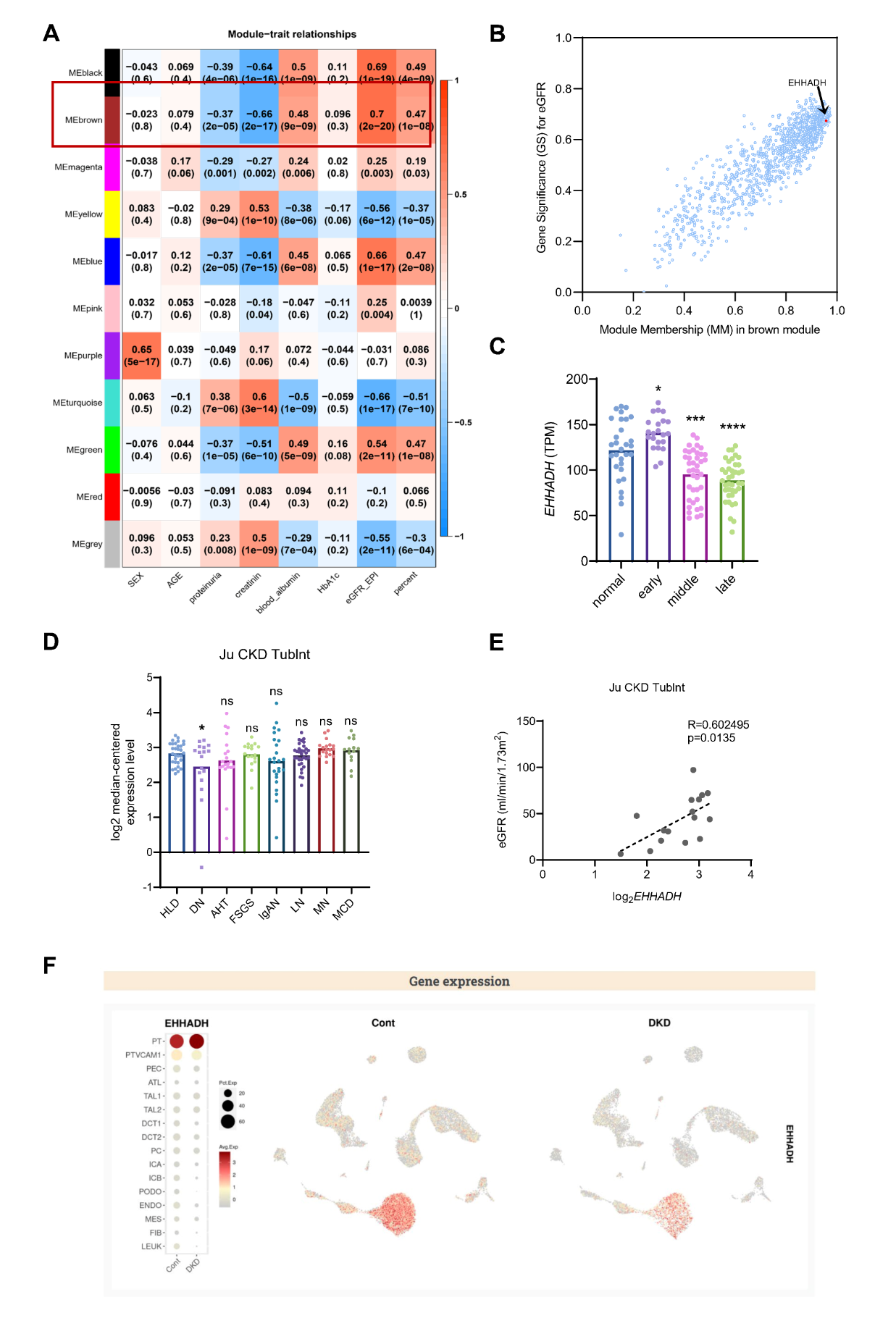


**Figure S2. Generation and validation of EHHADH-KO mice.** (**A**) EHHADH knockout strategy. Exons 2-5 were deleted with CRISPR/Cas9. (**B**) Representative images of IF staining of EHHADH in renal tubular cells of WT and EHHADH KO mice. **(C)** Western blotting analysis with kidney tissues from WT and EHHADH KO mice. **(D)** Quantification of the relative protein expression levels of Ehhadh. Two-tailed Student’s t test, **p < 0.01, ns: not significant. Values are the means ± SDs.


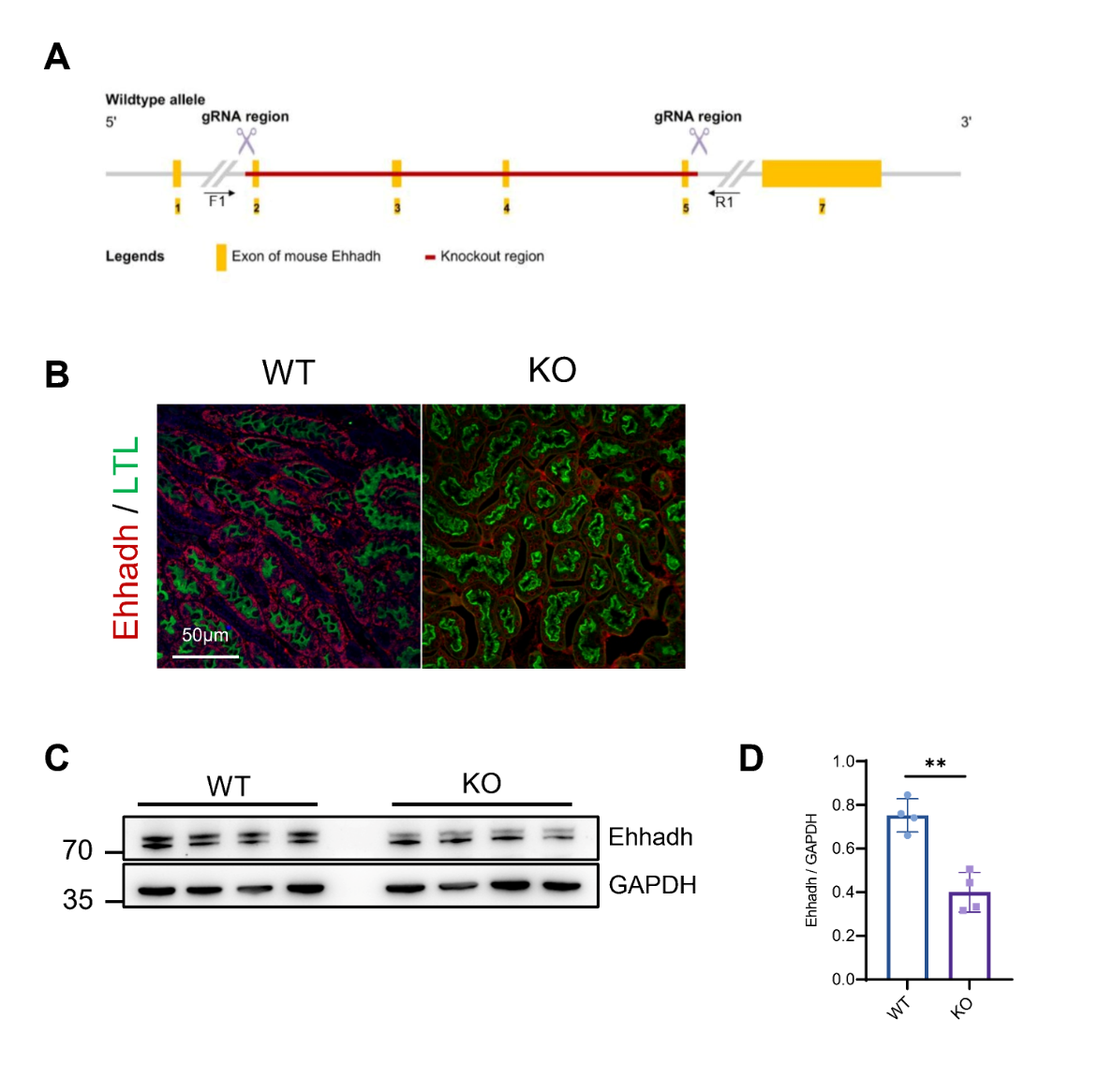


**Figure S3. High glucose treatment resulted in the decreased expression level of Ehhadh in NRK-52E cells. (A)** NRK-52E cells were treated with 24 h or 48 h of 55mM D-glucose (HG) and then harvested and analyzed by western blotting with the indicated antibodies. Mannitol (Man) was used as osmotic control. **(B)** Quantification of the relative protein expression levels of Ehhadh. Two-tailed Student’s t test, *p < 0.05, ns: not significant. Values are the means ± SDs.


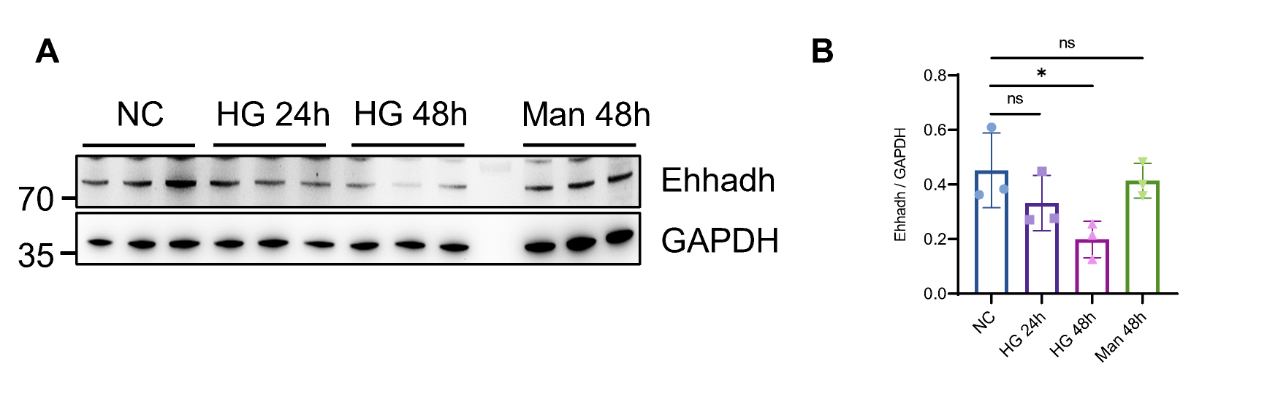


**Figure S4. EHHADH-mediated loss of peroxisomes was caused by enhanced pexophagy.** (**A**) NRK-52E cells were transfected with siNC or siEHHADH. After 24 h, the cells were treated with BzF (10 μM) for 24 h and then harvested and analyzed by western blotting with the indicated antibodies. (**B-E**) Quantification of the relative protein expression levels of Ehhadh, PMP70, PEX14 and PPARα. Two-tailed Student’s t test, *p < 0.05, **p < 0.01, ns: not significant. Values are the means ± SDs. (**F**) Immunofluorescence staining of PEX14 in NRK-52E cells transfected with si*NC* or si*Ehhadh*. (**G**) Quantification of PEX14-positive peroxisomes per cell. (**H**) Immunofluorescence staining of PEX14 and LAMP2 in NRK-52E cells transfected with si*NC* or si*Ehhadh* for 48 h and treated with or without 3-MA or BafA1. **(I)** Representative images of IF staining of PMP70 and LC3B in kidneys from control and diabetic mice. Two-tailed Student’s t test, *p < 0.05, **p < 0.01, ***p < 0.001, ****p < 0.0001. Values are the means ± SDs.


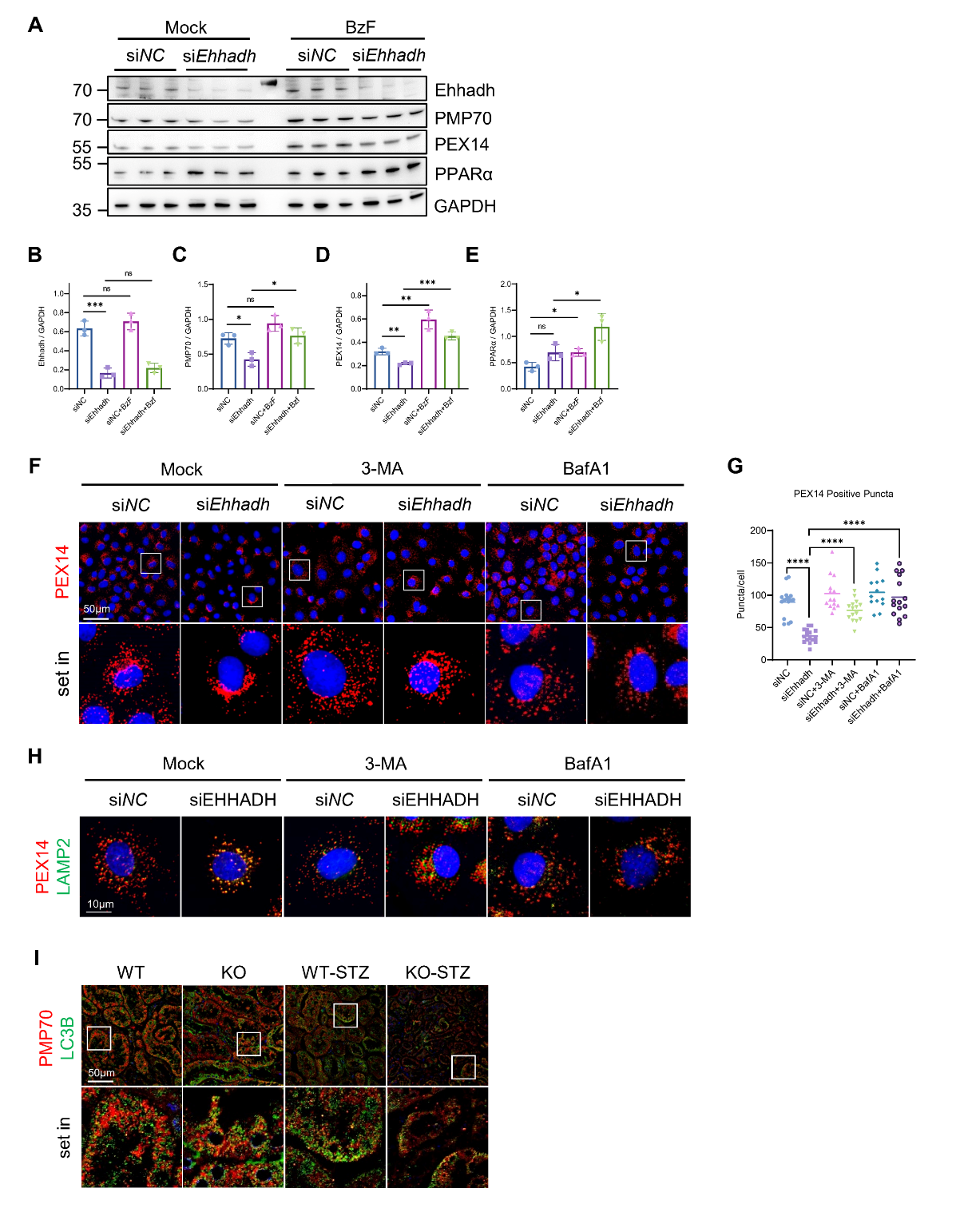


**Figure S5. Expression of NBR1 in EHHADH-knockdown cells.** (**A**) NRK-52E cells were transfected with siNC or siEHHADH, and after 48 h, the cells were harvested and analyzed by western blotting with the indicated antibodies. (**B**) Quantification of the relative protein expression levels of NBR1. (**C**) HEK293T cells were transfected with scramble siRNA (si*NC*) or *Ehhadh*-targeting siRNA (si*Ehhadh*), and the cells were harvested and analyzed by western blotting with the indicated antibodies after 48 h of transfection. (**D**) Quantification of the relative protein expression levels of EHHADH. (**E**) Immunoblots of lysates immunoprecipitated with anti-Myc magnetic beads from HEK293T cells 24 h after transfection with Myc-tagged PMP70 and HA-tagged K63-Ubi in the presence of si*NC* or *EHHADH*-targeting siRNA (si*EHHADH*) and then immunoblotted with the indicated antibodies. Two-tailed Student’s t test, *p < 0.05, **p < 0.01. Values are the means ± SDs.


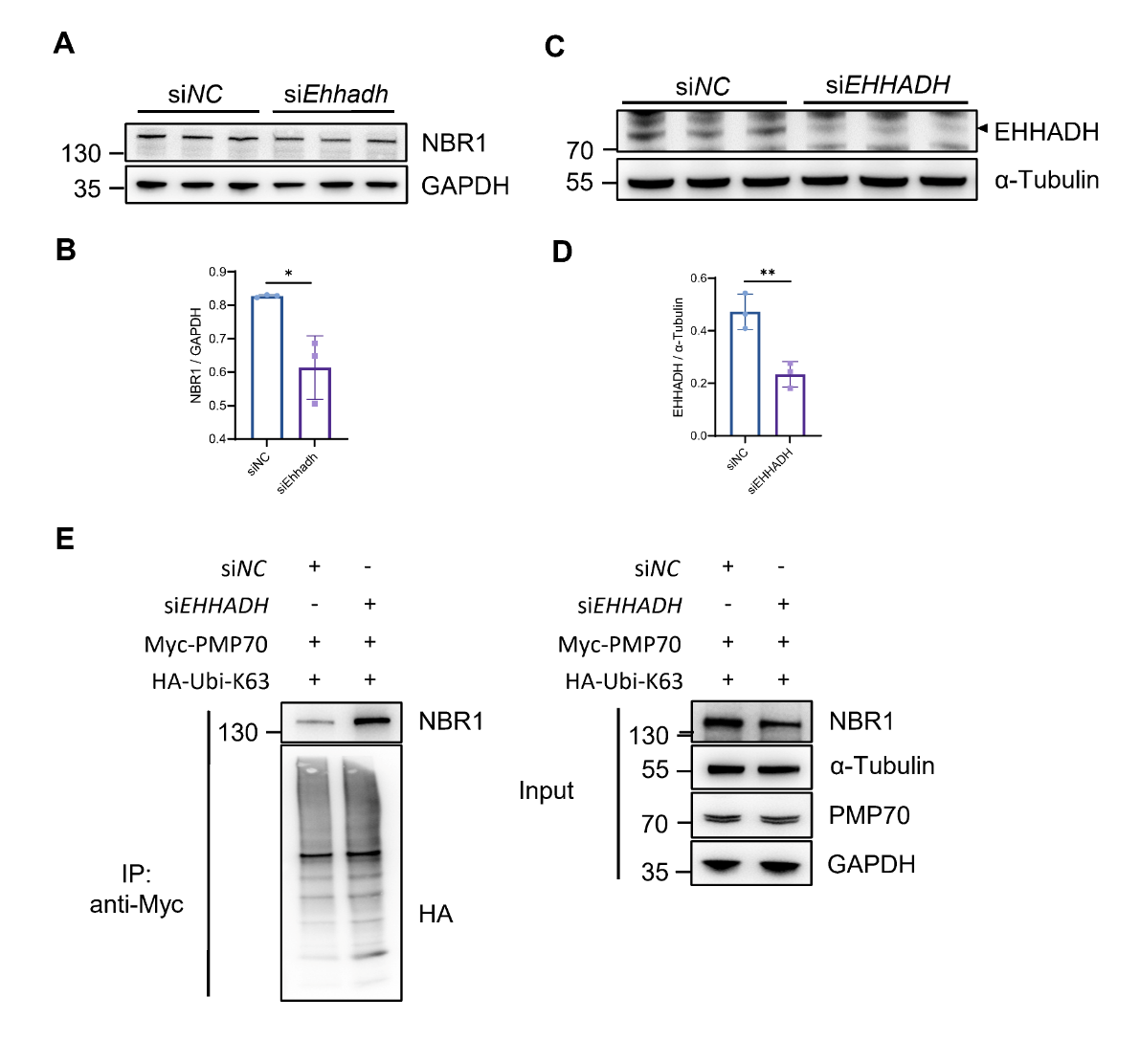

Supplement: Supplementary file 1 — Supplemental Figures [file 41420_2024_2066_MOESM1_ESM.docx]
