# Supplementary material for: EHHADH deficiency regulates pexophagy and accelerates tubulointerstitial injury in diabetic kidney disease
Source: Cell Death Discov. 2024 Jun 15;10:289. doi: 10.1038/s41420-024-02066-4 (PMC11180138; doi:10.1038/s41420-024-02066-4)
Supplement: Supplementary file 4 — Original data of WB [file 41420_2024_2066_MOESM4_ESM.pdf]

Fig. 3

A

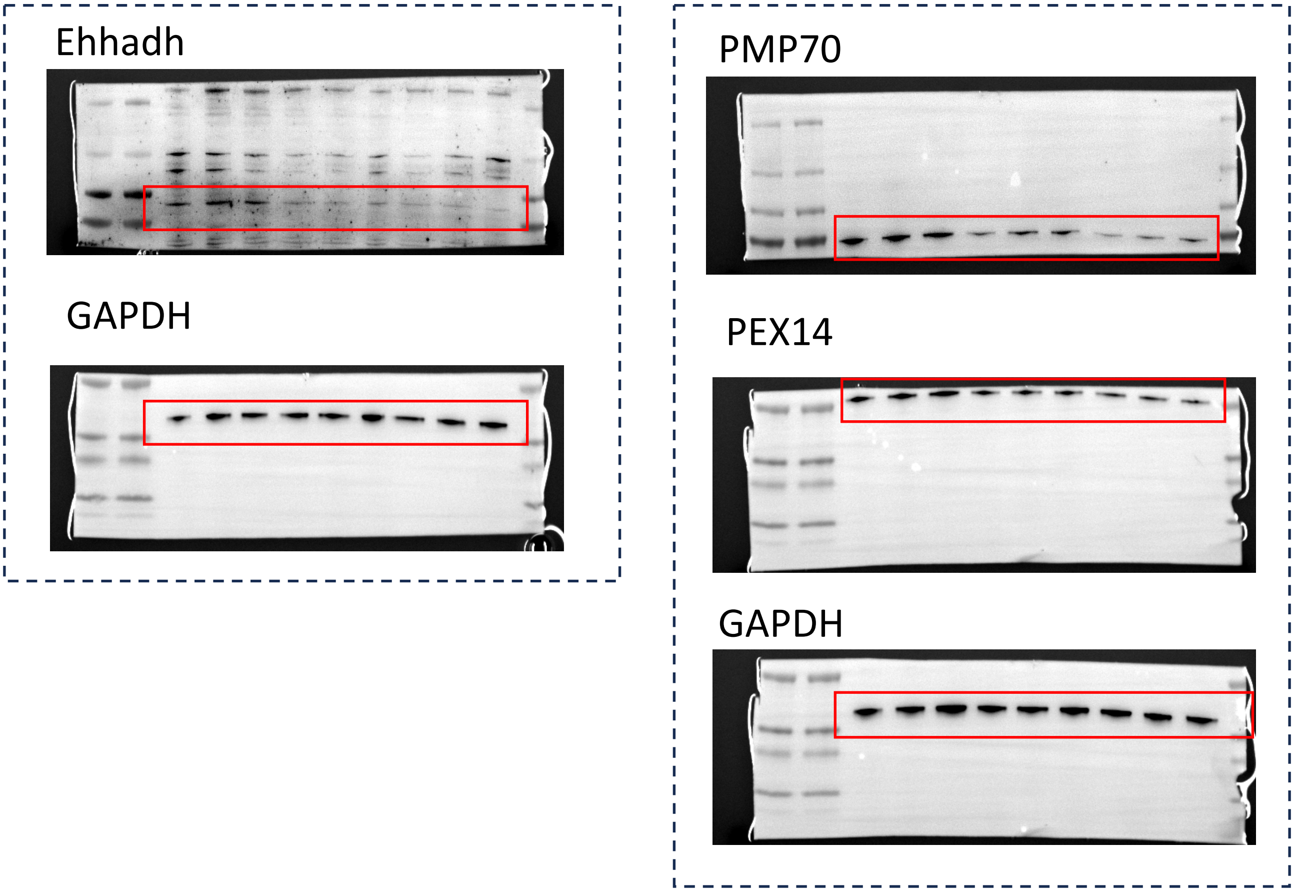

Fig. 4

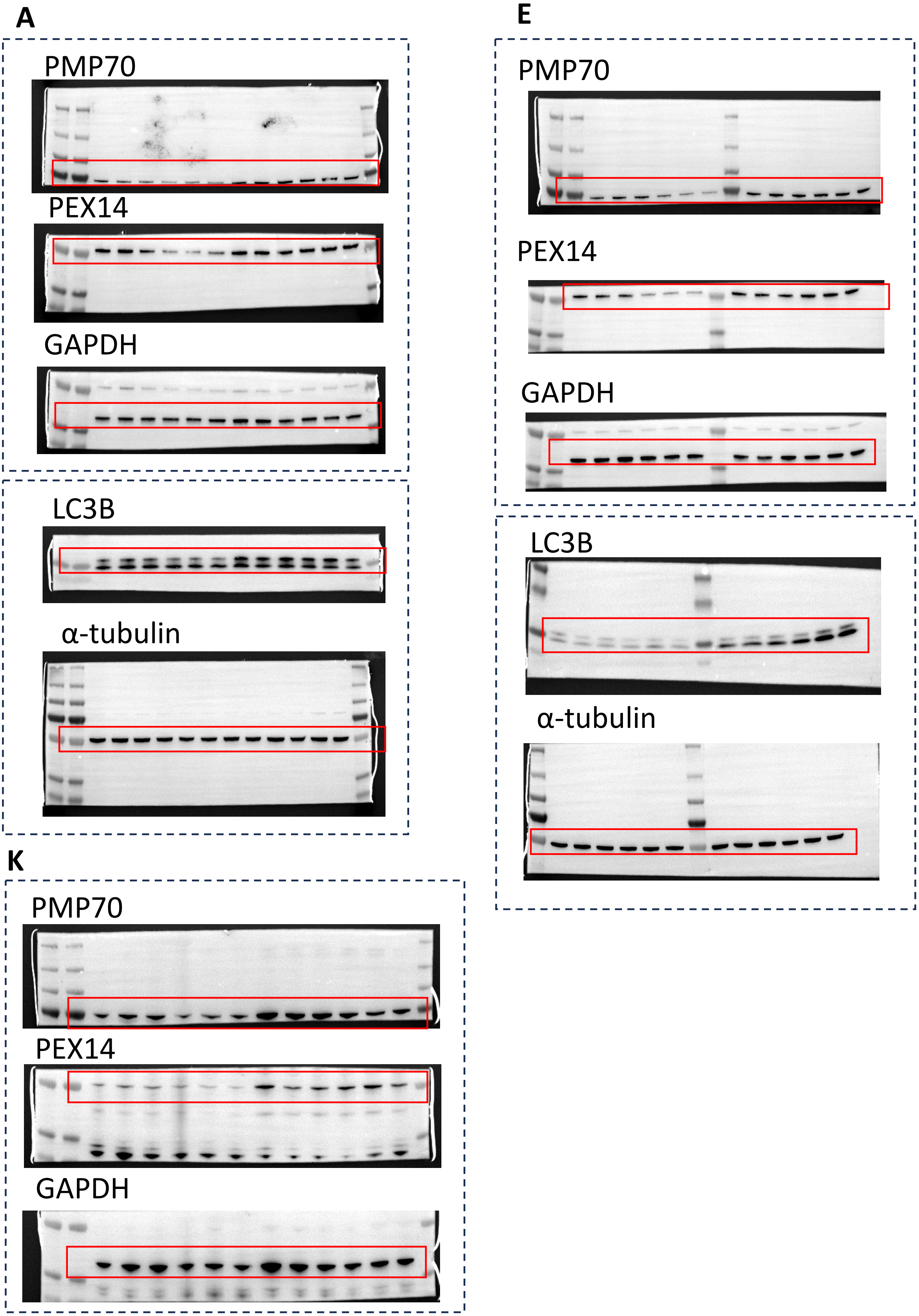

A
PMP70
PEX14
GAPDH
LC3B
α-tubulin
E
PMP70
PEX14
GAPDH
LC3B
α-tubulin
K
PMP70
PEX14
GAPDH

Fig. 5

C

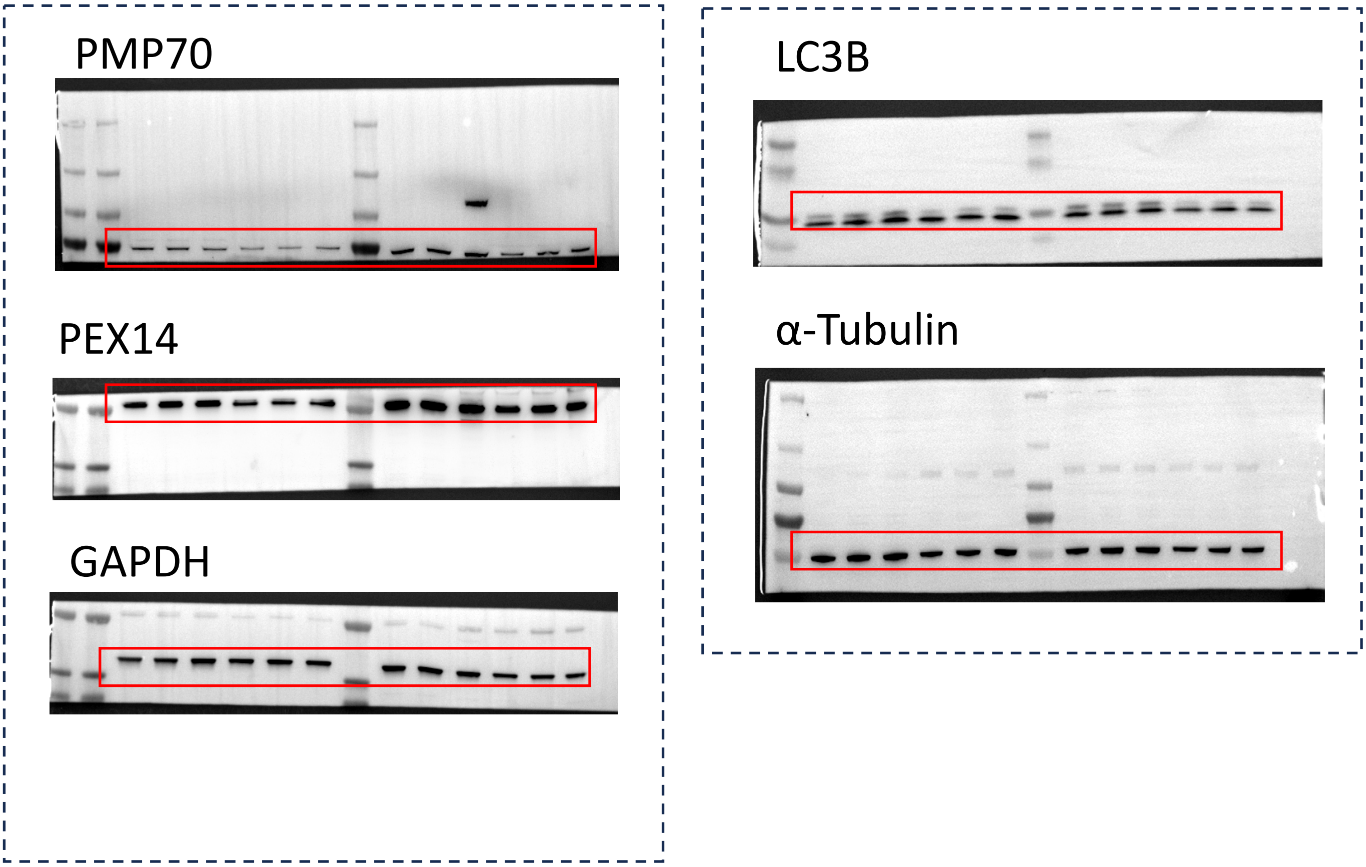

Fig. 6

A

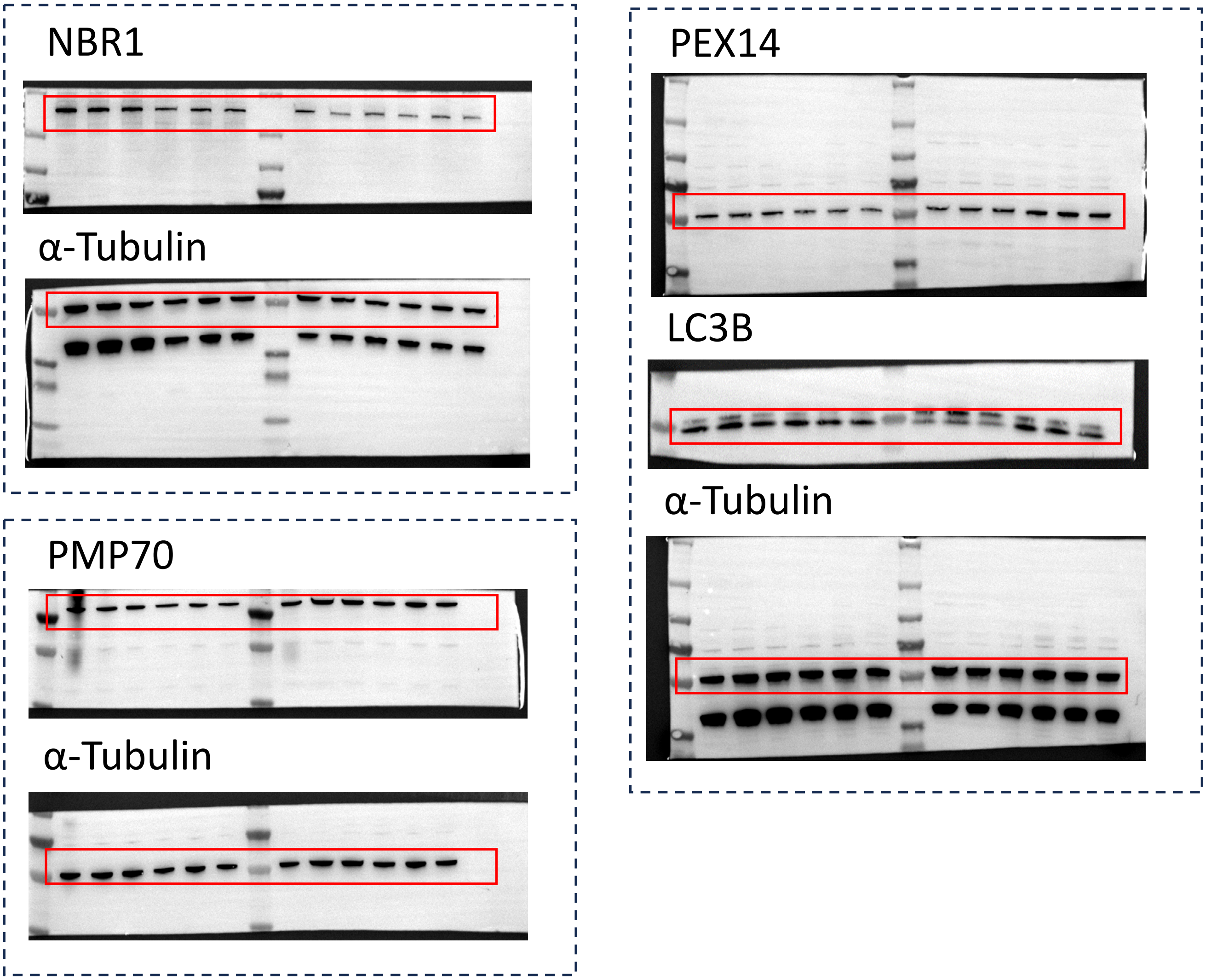

Figure S3

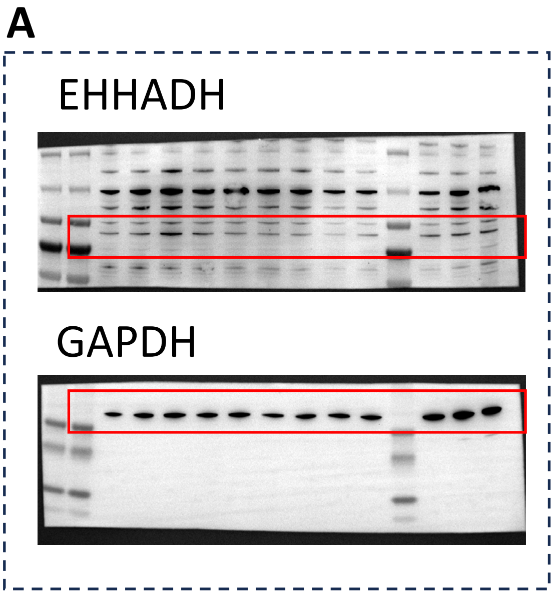

Figure S4

A

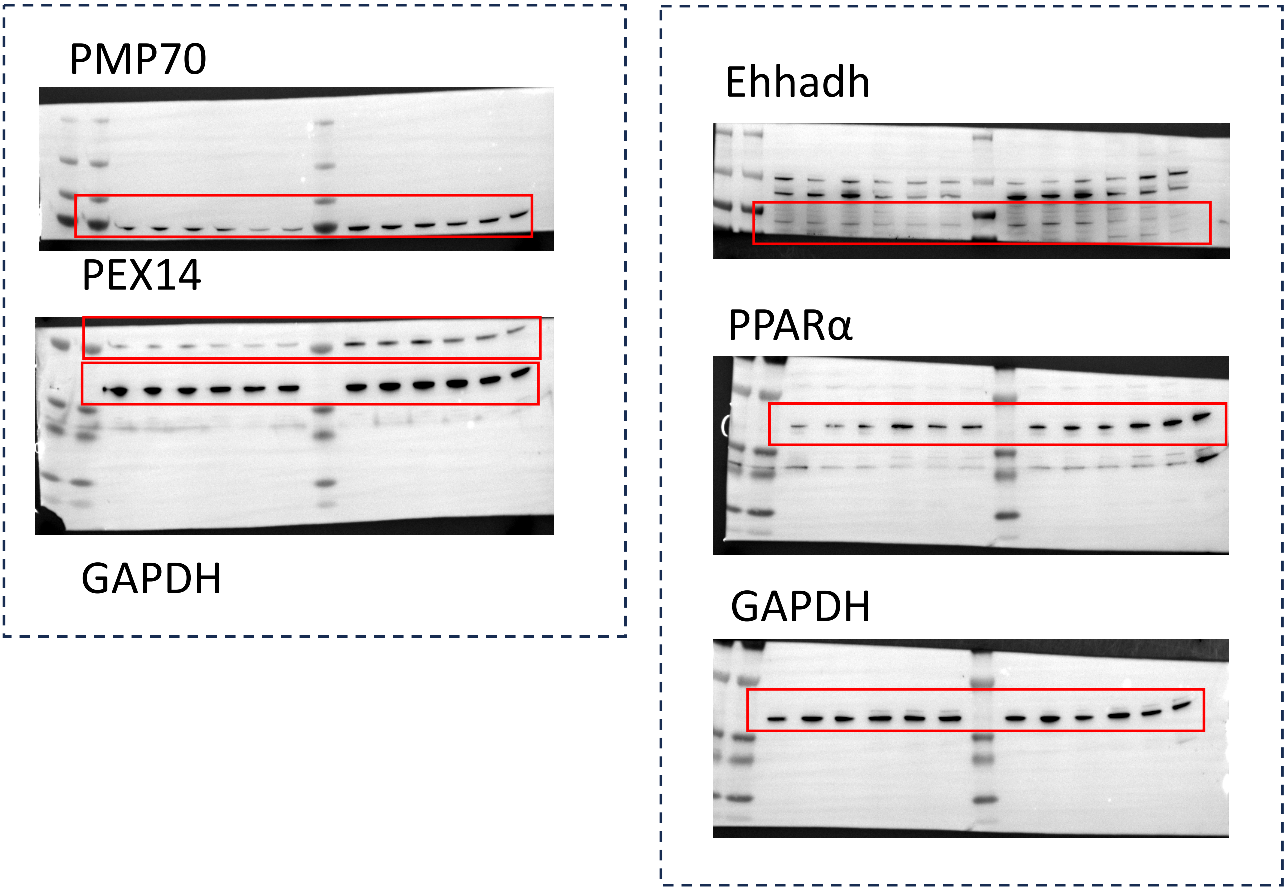

Figure S5

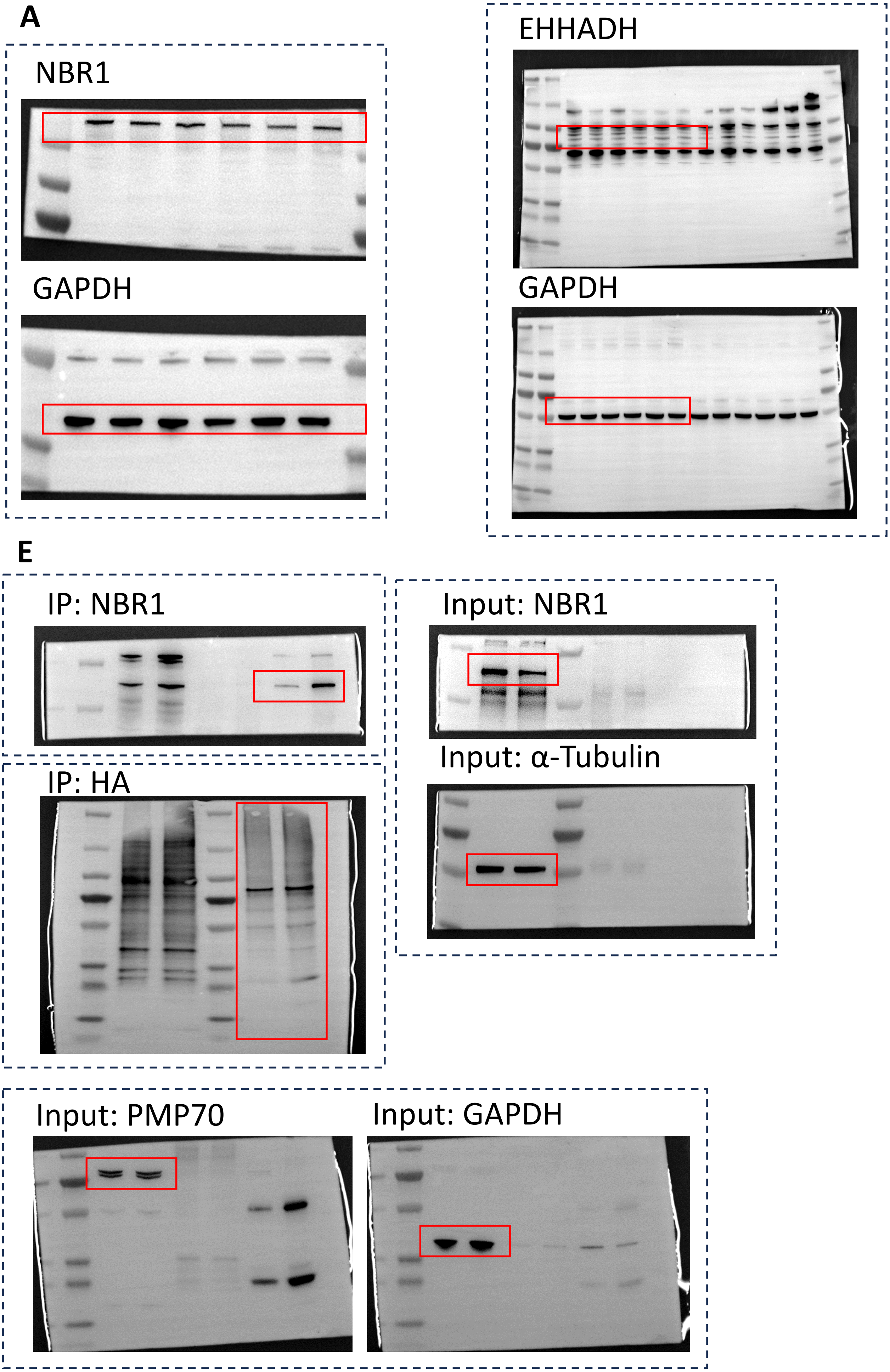
A
NBR1
GAPDH
C
EHHADH
GAPDH
E
IP: NBR1
IP: HA
Input: NBR1
Input: α-Tubulin
Input: PMP70
Input: GAPDH
